# Nitrate application or P deficiency induce a decline in *Medicago truncatula* N_2_-fixation by similar changes in the nodule transcriptome

**DOI:** 10.1038/srep46264

**Published:** 2017-04-10

**Authors:** Rebecca Liese, Joachim Schulze, Ricardo A. Cabeza

**Affiliations:** 1 Department of Crop Science, Section for Plant Nutrition and Crop Physiology, Faculty of Agriculture, University of Goettingen, Carl-Sprengel-Weg 1, 37075 Goettingen, Germany; 2 Departmento de Producción Agrícola, Facultad de Ciencias Agrarias, Universidad de Talca, Casilla 747, Talca, Chile

## Abstract

Nitrogen fixation of *Medicago truncatula* is regulated by the nitrogen status of leaves through inducing a repeatedly occurring 24-h nodule activity rhythm that reduces per day nitrogen fixation. The hypotheses of the present study were that (1) long-term moderate whole-plant P deficiency in *Medicago truncatula* induces an according daily rhythm in nitrogenase activity comparable to that induced by nitrate application and (2), the changes in the nodule transcriptome that go along with a strong nitrogenase activity decline during the afternoon would be similar under P deficiency or after nitrate supply. The nodules of plants in a low P treatment developed a rhythmic pattern of activity that resembled the pattern following nitrate application. A comprehensive, RNAseq-based comparative transcriptome profiling of nodules during a repeated part of the rhythm revealed similarities between P deficiency *versus* nitrate supply. Under both treatments, the formation of nitrogenase was targeted by a reduction in the expression of genes for nodule-specific cysteine-rich peptides (NCR), and possibly also by a disturbance of the inner cell iron allocation. A strong reduction in the expression of leghemoglobin is likely to have restricted the supply of oxygen for respiration.

Symbiotic nitrogen fixation is a cornerstone of sustainability in worldwide agriculture, but is a demanding process for legume plants, both with regard to assimilate and nutrient needs for nodule formation and also in terms of energy and carbon expenditure for the N_2_ reduction process. Estimates based on detailed measurements of the carbon balance in a greenhouse experiment reveal the energy cost of driving nitrogenase to be approximately equivalent to 25% of the shoot dry matter at harvest^1^. The plants therefore finely tune the amount of fixed nitrogen to the N demand of the shoots to minimise the energy costs for nitrogen fixation. To meet this challenge, the higher plant governs the symbiotic system by following two basic rules. Firstly, the legume plant uses any alternative N source preferentially, and secondly, when none is available, restricts nodule activity to the minimum necessary for sufficient growth. This is evident from the long-standing experience and experimental observation that plants with an ample supply of easily available N sources are superior in terms of dry matter production and nitrogen concentration when compared to plants that solely depend on nodules for N supply^2^.

The legume plant has two main mechanisms for applying the above basic rules for the symbioses. The first is regulation of the number of nodules and thus the available ‘machinery’ for symbiotic N_2_ reduction. The second is the ability to reduce nitrogen fixation of existing nodules quickly and efficiently if any alternative N source becomes available or any environmental cue restricts growth. The principal steps forming the molecular basis for the tight regulation of the nodule number are widely understood^3^,^4^,^5^. A root-shoot-root signalling system prevents hypernodulation (autoregulation) and is probably involved in adjusting the formation of additional nodules when growing shoots have an increasing N need^6^,^7^. In contrast, there is less understanding of the molecular processes by which existing nodules are downregulated when the N need decreases. Numerous studies have shown that nodules lose activity quickly (in hours to days) when alternative N sources became available or growth slows (caused for example by emerging nutrient deficiency)^8^,^9^,^10^. The basic principle appears to be a systemic N feedback regulation^11^,^12^. This means that a loss of nodule activity occurs when the plant leaves encounter available N beyond their requirement for the current or possible growth rate. This situation can be induced by various and diverse factors (emerging external or internal [senescing leaves] alternative N sources, restriction of growth). Under such conditions the nodules rather than showing a linear decline develop a 24-h activity rhythm that reduces the per day nitrogen fixation^13^. The continuous measurement of nitrogen fixation revealed that the daily pattern is apparently the primary reaction of nodule activity to various factors that have in common that the leaf N need is satisfied^13^. One consistently occurring part of the 24-h activity rhythm was what might be called an ‘afternoon decline’. A strong decline in activity began at around noon and abated and turned into an increase in activity at around 5 pm. One pronounced downregulation of nitrogen fixation activity of existing nodules is known to occur when nitrate becomes available^14^,^15^. The molecular basis for this phenomenon has been shown to consist of various molecular effects that rather than primarily restricting some external supply of carbon or oxygen, initially target nitrogenase formation and respiration on the transcriptional level^16^.

Based on the striking similarity of the pattern in the 24-h rhythm of nodule activity between nitrate application and other treatments that reduce nitrogenase activity^10^,^13^, the hypotheses of the present study were that (1) long-term moderate whole-plant P deficiency in *Medicago truncatula* induces an according daily rhythm in nitrogenase activity comparable to that induced by nitrate application and (2), the molecular reason for a strong nitrogenase activity decline during the afternoon – a major part of the daily rhythm – would be similar under P deficiency or after nitrate supply.

To validate these hypotheses, a procedure was established to grow *M. truncatula* plants in nutrient solution with limited P supply to produce lower growth but keep the plants alive. The growth system was embedded in a measurement system that allowed continuous, non-invasive monitoring of nitrogenase activity by measuring nodule H_2_ evolution^13^. In a further step the molecular reasons for the ‘afternoon decline’ induced by nitrate application or P deficiency should be revealed through a comprehensive RNAseq-based transcriptome profiling of nodules during that decline.

## Results

### Plant growth and P supply

**Figure 1:**
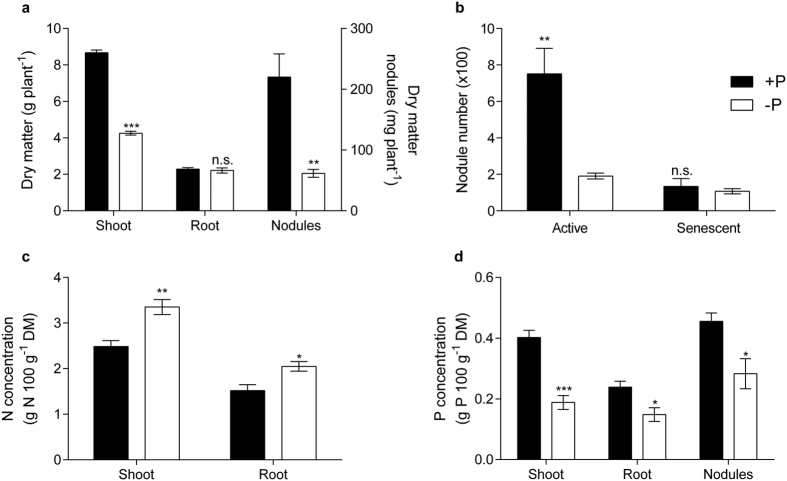
Dry matter formation and P and N concentration in eight-week old *M. truncatula* plants grown at sufficient and limited P supply. Plants were grown with a daily adjustment of the nutrient solution to either 5 μM or 1 μM P in the sufficient (+P) and limited (−P) P-treatment respectively. The P concentration in the +P treatment was never reduced below 3 μM by the plants during the experiment, while the concentration in the limited P treatment reached the C_Lmin_ value every day. Consequently while the plants in the +P treatment enjoyed a continuous P supply, the plants in the -P treatments suffered limited supply. The leaves of the -P plants partially developed a reddish colour, known to be the result of anthocyanin formation^48^, a symptom of P deficiency^49^. At the same time, a particular deep green colour of the remaining parts of the leaves was indicative of the higher leaf N concentration in the -P treatment. Data are means of six replicates. The two treatments were compared by the t-test. Statistical differences in comparison to the +P treatment are indicated by stars (*) above the bars (*different with a probability of 95%, **different with a probability of 99%, ***different with a probability of 99.9%; n = 6; n.s. = not significantly different).

Plants of *M. truncatula* previously inoculated with *Sinorhizobium meliloti* (102F51) and with visible active nodules were grown in a quasi flow-through nutrient solution system, where the P concentration was adjusted daily. Using this system, a daily adjustment of the P concentration to 5 μM proved optimal for growth of *M. truncatula* when depending solely on nitrogen fixation for N supply. A daily measurement of the P concentration before the adjustment to 5 μM showed that the concentration was never depleted below 3 μM P during the experiment (eight weeks of growth), thus allowing the plants a continuous optimal P uptake and N_2_-fixation. As a treatment with limited P supply (P deficiency), a daily adjustment of the nutrient solution to 1 μM (20% of full P supply) was chosen. The daily P concentration before the adjustment of the solution showed that the plants reduced the concentration to a minimum value below which the plants cannot take up P anymore^17^ (C_Lmin_ value [0.2–0.3 μM]) throughout the experiment and thus suffered a limited P supply. The preliminary experiment had shown that *M. truncatula* plants under these conditions experienced reduced growth, but maintained a certain level of nitrogen fixation and did not die during the planned eight-week experimental period. Dry matter (DM) formation, nodule number and N and P concentrations in the various organs of the plants are shown in Fig. 1. The plants of the fully nourished treatment showed vigorous growth in these experiments and developed more than 10 g DM during the eight-week experimental period (Fig. 1A). The DM of shoots, nodules and the shoot/root ratio were significantly reduced by the low P treatment. Active nodules were significantly lower in P-deficient plants and the number of senescent nodules was not different for both treatments (Fig. 1B). N concentration in shoots and roots was significantly higher in the low P treatment (Fig. 1C) and the nodule P concentration in the low P treatment was lower when compared to the nodules of the control (Fig. 1D). The relative reduction in shoot P concentration was stronger than that in nodules. The overall picture with respect to these parameters was therefore in accordance with moderate P deficiency (slower growth, reduced shoot/root ratio and increased N concentration in leaves^18^,^19^,^20^,^21^).

### Nitrogen fixation under low P

**Figure 2:**
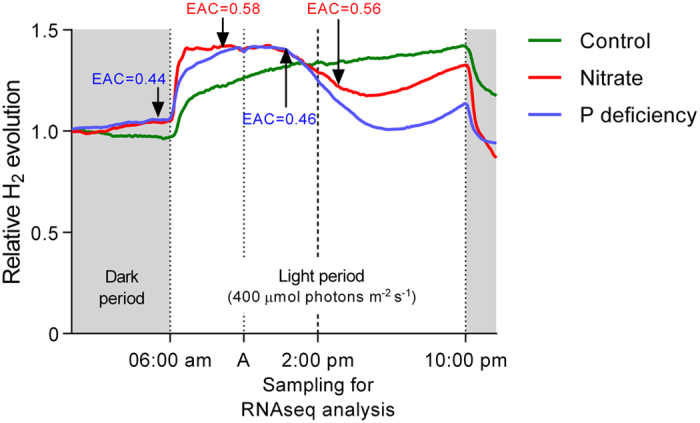
Whole-plant H_2_ evolution (nitrogen fixation) over a 24-h period of nodulated *M. truncatula* plants in a fully nourished control, during nitrate application and after 8 weeks of growth with limited P supply. The time course of nitrogen fixation in the control (green line) during nitrate application (red line) and after 8 weeks of growth under limited P supply (blue line) is shown during the 16-h light period (white background) and 8 h of darkness (grey background). Nitrate was applied at the point in time indicated by the dotted line labelled with A. At 2:00 pm, nodules were harvested for the RNAseq comparative transcriptome analysis. Measurements of the electron allocation coefficient (EAC = relative efficiency of nitrogen fixation) showed no shifts within a treatment over the time period, but a lower EAC in the P deficiency treatment when compared to the control and the nitrate application treatment. Data were taken every 5 min as means of six replicates. Data for control and nitrate were taken from^16^, www.plantphysiol.org, Copyright American Society of Plant Biologists.

Nitrogen fixation was measured continuously during one day in the eighth week of growth. While the plants in the control showed a stable nitrogen fixation throughout a 24-h period, the low-P plants had developed the typical daily rhythm. In particular, a strong decline occurred during the time between about noon and 5 pm (Fig. 2). Nitrogen fixation efficiency was reduced under low-P conditions. A lower relative efficiency (electron allocation efficiency [EAC]) compared to the control plants (Fig. 2) contributed to that. The low EAC did not change when measured at noon compared to measurement after eight hours of darkness during the night. Furthermore, the nodule-specific activity, expressed as μg N fixed per day and mg nodule DM (active nodules), was about 35% lower when compared to the control plants (Table 1). Active nodules were distinguished from inactive nodules by a pinkish *versus* a greenish to brown colour of the nodules. Although we are not aware of any direct measurement that greenish nodules have totally lost their nitrogenase activity, the fact that the green colour results from a decay product of leghemoglobins, which are of vital importance in nodule functioning, makes this highly probable. In any event, when considering the entirety of the collected nodules, the gap between the control and low-P treatment would even have widened since the share of inactive nodules was higher in low-P plants.

### Gene expression after nitrate application (Mt3.5v3 versus Mt4.0v1)

The study was supported by the fact that the harvest of P-limited nodules for RNAseq analysis was performed at the exact date and time as that of the nitrate-treated plants and the control^16^. The RNAseq experiment was performed before the study’s authors became aware of the importance of the repeated 24-h rhythm in nodule activity under treatments that reduce nitrogen fixation. The original reason for combining both experiments was to be able to use one control for both treatments (nitrate application and long-term P deficiency). However, the fact that both treatments were grown in the same experiment and harvested at the same age and time of day also allowed a valid comparison of the transcriptome of nodules from nitrate-treated plants *versus* those from plants grown under limited P supply. The data of the comparison nitrate versus control are published in Cabeza *et al*.^16^ (www.plantphysiol.org, Copyright American Society of Plant Biologists), but were recalculated on the basis of the newly released, significantly enhanced cds annotation for *M. truncatula* (Mt4.0v1 *versus* Mt3.5v3 that was previously used). The reason for that approach was to form a basis for comparison of the molecular reasons for the ‘afternoon decline’ induced by long-term P deficiency versus nitrate application. The harvest in both treatments was done at the exact same time, at which in both cases the ‘afternoon decline’ was in full effect (Fig. 2).

The main described effects of nitrate on the nodule transcriptome^16^ were confirmed and reinforced (for example by the number of downregulated Transcription Units [TU] for nodule-specific cysteine-rich [NCR] peptides) by revisiting the data with the new cds annotation (Supplementary Dataset File 1: sheet 2). A strong downregulation of numerous genes for NCR-peptides was confirmed along with the downregulation of all expressed genes for leghemoglobin. Furthermore, a putative effect on nodule iron turnover was confirmed. In particular, a strikingly strong downregulation of a gene for a ‘nicotianamine synthase-like protein’ (Medtr1g084050) (log_2_FC = −5.0) was again found with the new cds annotation. The importance of the gene for efficient nitrogen fixation and for nodule acclimation to altered external oxygen pressure has recently been shown^22^.

### Gene expression in nodules from plants with moderate P deficiency

**Figure 3:**
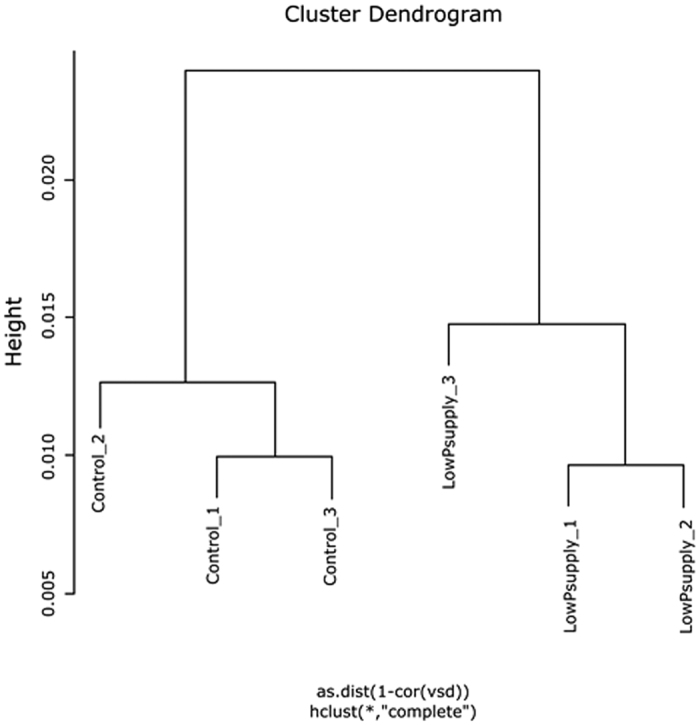
Dendrogram for RNAseq analyses of control and -P nodules. Replicates were similar within the control and treatment, while the treatment replicates were different from those of the control (n = 3). The cluster analysis was calculated with the R package “hclust” through a complete linkage clustering.

Nodules for the RNAseq analysis were harvested during the P deficiency induced ‘afternoon decline’ parallel with the nodules harvested 4 h after nitrate application (see Fig. 2). The sequencing yielded 10 to 15 million mapping reads per sample and thus a sufficient depth for the objective of our analysis. About 80% of the reads hit at least one time in the cds annotation Mt 4.0v1. About 33,000 TUs of the cds annotation were hit at least one time. When setting a threshold of >20 DESeq normalised counts in either control, treatment or both (see Supplementary Dataset File 2: sheet 1) 16,725 TUs were found to be expressed. This corresponded to 26.8% of all TUs annotated in the cds annotation Mt4.0v1. A cluster analysis (Fig. 3) shows that within the control and P-deficiency treatment, the replicates resembled one another, while there was a clear difference between the treatment and the control. A DESeq comparison of the control and the treatment revealed 1,738 differentially abundant TUs (False Discovery Rate [FDR] < 0.01; n = 3) which represent a 10.4% of the expressed TUs (Supplementary Dataset File 2: sheet 2). Of these TUs 900 were differentially expressed with a log_2_FC greater than [1], meaning that the abundance of the mRNA was at least doubled or halved. A quantitative reverse transcription-polymerase chain reaction (qRT-PCR) validation proved the reliability of the RNAseq data (Supplementary Dataset File 2: sheet 5). Twelve genes were tested using the same RNA pools previously used for the next-generation sequencing. The qRT-PCR results were significantly correlated to the RNAseq data (r^2^ = 0.89). The slope of the regression line was close to one (0.94).

## Discussion

The low-P treatment resulted in P-deficient plants that could be kept alive during eight weeks of growth in the present study’s experimental system. The plants showed typical growth reactions to limited P supply^21^,^23^. A higher N concentration in the shoots supports the view that plant growth under these conditions is not limited by nitrogen fixation but rather restricts nodule activity, possibly by a shoot-related N-feedback effect^20^,^24^. The low-P treatment in this experiment resulted in N_2_-fixation that clearly showed a 24-h rhythm of nitrogenase activity. The pattern of the rhythm was consistent with that under other treatments^13^. The relative, specific and per plant activity of the nodules under P deficiency was significantly lower when compared with the control plants. There are scattered reports that nodule-specific activity remains unchanged or is even increased under low P^20^,^21^. This was clearly not the case under the present study’s conditions, even when only undoubtedly active nodules (pink colour) were considered. Overall, the specific activity might be maintained depending on the age of the plants and the extent of the P deficiency^19^,^25^, but is generally lower under whole-plant P deficiency. In addition, strong variations in measurements of specific activity might have been the result of point measurements at different times during the 24-h activity cycle. For example, under our conditions a point measurement in the P-deficient treatment at 5 pm would have yielded a value about one third lower when compared to a corresponding measurement at noon.

**Figure 4:**
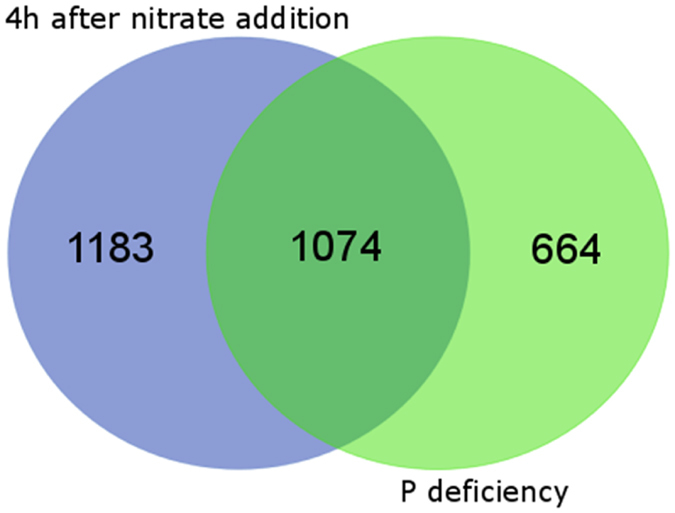
Venn diagram showing the number of differentially expressed TUs in nodules 4 h after nitrate provision or after 8 weeks of limited P supply. Data are the result of a DESeq analysis of RNAseq counts derived from RNA of nodules in three biological replicates. The reads were aligned to the cds annotation Mt4.0v1. The count number was considered significantly altered from the number in the control when the FDR value was below 0.01.

**Figure 5:**
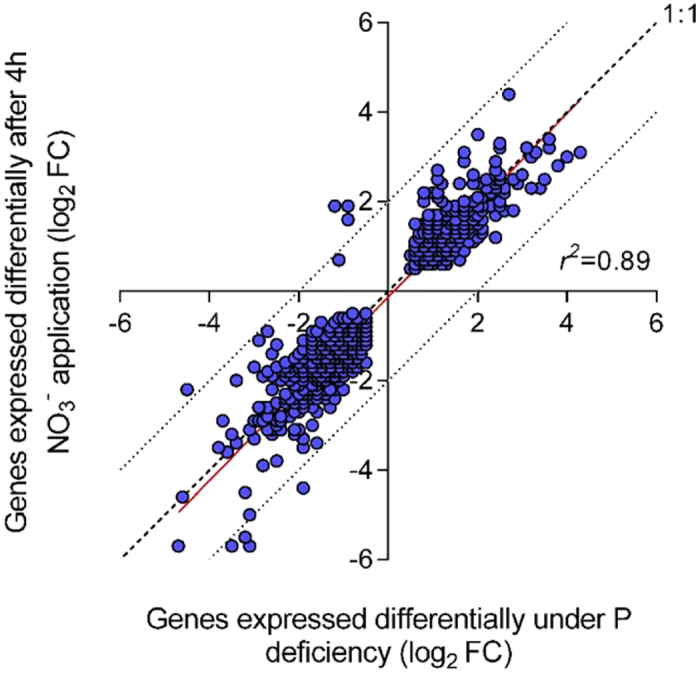
Scatter plot of common TUs differentially abundant after 4-h nitrate impact or after 8 weeks of limiting P supply. Data are the result of a DESeq analysis of RNAseq counts derived from RNA of nodules in three biological replicates. The reads were aligned to the cds annotation Mt4.0v1. The count number was considered significantly altered from the number in the control when the FDR value was below 0.01.

Comprehensive nodule transcriptome profiling based on RNAseq analysis revealed that during the ‘afternoon decline’ of the 24-h activity pattern, similar molecular mechanisms appeared to be active in nodules of treatments as diverse as low-P supply *versus* (over)-sufficient nitrate application (Supplementary Dataset Files 3 and 4). In a sense, P deficiency and external N supply (nitrate) can be considered as the most distinct treatments that reduce nitrogenase activity. While one is promoting plant growth (nitrate) and is known to reduce nodule oxygen permeability, moderate P deficiency reduces plant growth and increases nodule oxygen permeability. Nevertheless, similar molecular mechanisms are active during the ‘afternoon decline’ of nodule activity. This was first indicated by the fact that while both treatments showed strong shifts in the transcriptome when compared to the common control (low-P or high nitrate, 1,738 or 2,257 TUs respectively; [FDR < 0.01, n = 3]), a direct comparison between the transcriptome of the nodules in both treatments revealed only 663 TUs to be of different abundance (FDR < 0.01, n = 3) (Supplementary Dataset File 3: sheet 2). That means that many of the changes in both treatments in comparison to the control went in the same direction. Among those still different 663 TUs in the direct comparison of P deficiency and nitrate application were 86 cases in which significant changes also went in the same direction compared to the control. However, a different extent of the change left significantly different TU abundances when the two treatments were compared. Of the remaining TUs, numerous are originated from genes with a known nitrate-specific regulation (nitrate transporters, nitrate and nitrite-reductase). When the lists of differentially regulated TUs in the two diverse treatments *versus* their common control were compared, an intersection of 1,074 TUs was found (Fig. 4). This corresponded to 48 or 62% of all differentially regulated TUs in the nitrate-treated or P-deficient nodules respectively. A closer look at the log_2_FC rates revealed a striking similarity between the reaction of the regulated TUs in the two diverse treatments, with a slightly stronger effect overall of the nitrate (log_2_FC average for all differentially expressed TUs either up or down regulated: nitrate +1.38, −1.48; P-def. +1.35, −1.25) (Supplementary Dataset File 4: sheet 1). The log_2_FC numbers showed a close correlation (r^2^ = 0.89) and the predicted regression line was close to a 1:1 correlation (Fig. 5).

The commonly regulated TUs of both treatments reflected all the effects described for nitrate^16^. When the log_2_FC of significantly and commonly regulated TUs of both treatments were weighted together by the RPKM (Reads Per Kilobase per Million mapped reads) values of the control (RPKM_control_ × log_2_FC after 4-h nitrate impact × log_2_FC after eight weeks of low P supply) the above-mentioned Medtr1g084050.1, annotated as ‘nicotianamine synthase-like protein’, was the most strongly affected TU (Supplementary Dataset File 4: sheet 2). Iron is of central importance for nodule functioning because it is an essential component of nitrogenase, leghemoglobin and ferredoxin^26^. Nicotianamine synthase (NAS) is of pivotal importance for the inner cell and inner plant allocation and re-allocation of iron and other metal ions^27^,^28^. Among the TUs annotated as NAS, Medtr1g084050.1 was the by far most strongly abundant in nodules (Supplementary Dataset File 4: sheet 3). It has recently been shown, partially on the basis of a *Tnt1* mutant, that this gene is important for nodule efficiency and the necessary neoformation of nitrogenase after the impact of elevated oxygen on nodules^22^. Taken together, it appears possible that the iron supply of bacteroids and infected cells might function as a regulatory mechanism for nitrogenase formation.

There were a total of 203 TUs for NCR-peptides that were downregulated under both treatments from a high level of expression in the nodules. In fact, when the log_2_FC in both treatments were weighted by the RPKM values of the control, TUs for NCR peptides made up 56 of the 100 most differentially regulated TUs. NCR peptides are formed in the nodules of legumes of the inverted repeat-lacking clade of legumes and targeted to bacteroids^29^. They are closely related to defensins and attach to the bacteria to keep them in the bacteroid stage. This stage basically means that the bacteria cease to divide and strongly reduce metabolic activity except that they form nitrogenase vastly in excess of their own needs^30^. The delivery of these peptides appears to be the way in which the higher plant forces (sanctions) the microsymbiont to return sufficient ammonia in exchange for energy and nutrients^31^,^32^. During the ‘afternoon decline’ under both treatments, this pressure was strongly reduced. Thus a concerted downregulation of the expression of numerous genes for these peptides appears to be among the initial mechanisms that induce the ‘afternoon decline’ in nodule activity. Recent reports show that even the loss of function of a single gene for an NCR peptide results in reduced nitrogen fixation. The mutants *dnf4* (defective in nitrogen fixation) and *dnf7* are impaired in the functioning of the gene Medtr4g035705 and Medtr7g029760 respectively. Both genes encode an NCR-peptide^33^,^34^ and were expressed strongly in the nodules in the present experiments (RPKM Medtr4g035705.1 and Medtr7g029760.1 = 445 and 230 respectively, see Supplementary Dataset File 2: sheet 4) and significantly downregulated by both treatments (log_2_FC Medtr4g035705.1 −0.8 or −0.59 under nitrate or P deficiency respectively; log_2_FC Medtr7g029760.1 −1.42 or −0.9 under nitrate or P deficiency respectively). Nevertheless, the expression and also the weighted log_2_FCs in transcript abundance of both TUs for the two NCR-peptides were only to a medium extent when considering the numerous other affected TUs for NCR-peptides. This fact might support the specific individual importance of the different NCR-peptides. On the other hand, in an earlier study, the individual knockdown or overexpression of five different genes for NCR-peptides resulted in no detectable phenotype, suggesting a certain redundancy among the NCR-peptides^35^. Our data suggest that the regulation of the NCR-peptides genes occurs in a concerted, one might say ‘module-like’-fashion. The targeting of the peptides to the symbiosome is ensured through a signal peptidase complex. A mutation in the gene Medtr3g027890 (*dnf1*), which encodes for a core unit of this complex, results in fix^−^ nodules^36^. Expression of the gene was not affected during the ‘afternoon decline’, however it was strongly reduced when the nitrate impact had lasted for 28 h and the nodules had lost almost all activity and showed molecular indications for senescence^16^.

A further clear common effect of the treatments was the concerted downregulation of almost all the expressed genes for TUs of leghemoglobin. Eleven of the 16 genes annotated as leghemoglobins in the cds annotation Mt4.0 v1 were expressed and nine of them were strongly downregulated by both treatments in the nodules in the present experiments (Supplementary Dataset File 4: sheet 3). The expression had reached a high level in the control. This could be illustrated by the fact that five of these TUs were among the 100 most strongly abundant TUs in the nodules, and is supported by leghemoglobin protein accumulating to mM concentrations in the cytoplasm of nodule cells^37^. Functionally, leghemoglobin provides a substantial buffer capacity for free oxygen in the nodule interior, contributing to the maintenance of free oxygen concentrations at a low nM level. Furthermore, the leghemoglobin protein ensures a constant flow of oxygen for the high-affinity bacterial cytochrome oxidases to maintain sufficient respiration. The two imposed treatments are known to have a different effect on nodule O_2_ conductance. While nitrate has been shown to lead to lower O_2_ influx into the nodule and lower partial oxygenation of leghemoglobin^38^, nodule O_2_ conductance increases under P deficiency^25^,^39^. The strong downregulation of leghemoglobin TUs under both treatments consequently suggests that O_2_ buffering is not the primary function of leghemoglobin. Instead the lower expression of leghemoglobins might be a mechanism for lowering nodule and bacteroid respiration and thus ATP production for driving nitrogenase. RNA_i_ interference with a gene for leghemoglobin in *Lotus japonicus* resulted in a fix^−^ phenotype^40^. The loss of function of the leghemoglobin gene was accompanied by an almost total loss of nitrogenase protein in the nodules. This finding is supported by the comparative transcriptome analyses data so far obtained in our lab, in that the concerted expression of all expressed leghemoglobin genes always accompanies the nitrogenase activity of the nodules and the expression of genes for NCR-peptides^10^,^16^,^22^. These observations support the view that leghemoglobins might have functions beyond O_2_ binding and buffering^41^, possibly in oxygen concentration signalling and regulating nodule activity.

In conclusion, the data from the present study supported the fact that the downregulation of nitrogenase under conditions of over-sufficient whole-plant N availability (induced by nitrate application or P deficiency) initially occurs through a 24-h rhythmic pattern of nodule activity. This pattern is induced by common molecular mechanisms in the nodule directly involved in nitrogenase formation and respiration.

## Materials and Methods

### Plant growth

Seeds of *Medicago truncatula* (Gaertn.) cv. Jemalong A17 were submerged in H_2_SO_4_ (96%) for 5 min for chemical scarification, sterilised with 5% (v/v) sodium hypochlorite for 3 min and rinsed several times with deionised water. The seeds were subsequently kept at 4 °C for 12 h in darkness, submerged in tap water. The next step was a two-to-four day slight shaking of the submerged seeds at 25 °C and continuous light. When the seeds had developed a *ca*. 20-mm-long primary root, they were transferred to small growth boxes (170 mm × 125 mm × 50 mm) filled with aerated nutrient solution. Each box contained 20 seedlings, which were fixed by small x-shaped cuts in the adhesive tape on the upper side of the boxes.

The plants were grown for two weeks in these boxes in a growth chamber with a 16/8-hour light/dark cycle at 25/18 °C respectively. The nutrient solution level in the boxes was maintained by the addition of an appropriate amount of nutrient solution every other day. The light intensity at plant height was approximately 400 μmol m^−2^ s^−1^. Immediately after being transferred to the growth boxes, the seedlings were inoculated with 1 mL box^−1^ of a stationary *Sinorhizobium meliloti* (Sm) (102F51) YEM-culture of an approximate cell density of 10^9^ mL^−1^. The Sm strain induced good nodulation with the first macroscopic nodules visible after about 7–10 days. This strain does not contain an uptake hydrogenase^42^.

After two weeks, the plants were transferred to glass tubes, allowing the separate measurement of root/nodule H_2_ and CO_2_ evolution. The system is described by Fischinger and Schulze^43^ and Fischinger *et al*.^44^. The setup was enhanced by connecting a group of six plants through the lower side of the glass tubes to a 20-L nutrient solution container. Thus, using gravity, the nutrient solution level in the glass tubes depended on the height of the container, and losses *via* plant transpiration could be adjusted by the addition of nutrient solution to the container, thereby not interfering with the measurements in the root/nodule compartment. In addition, a pump in the container drove a nutrient solution flow of about 10 mL min^−1^ into the upper part of each individual glass tube^13^. Each tube contained about 150 mL nutrient solution, which was replaced every 15 min. The nutrient solution in each glass tube was individually aerated with a gas stream of 200 mL min^−1^ (N_2_/O_2_; 80/20; v/v). The nutrient solution contained macronutrients (mM) K_2_SO_4_, 0.7; MgSO_4_, 0.5; CaCl_2_, 0.8 and micronutrients (μM) H_3_BO_3_, 4.0; Na_2_MoO_4_, 0.1; ZnSO_4_, 1.0; MnCl_2_, 2.0; CoCl_2_, 0.2; CuCl_2_, 1.0 and FeNaEDTA (ferric monosodium salt of ethylenediamine tetraacetic acid), 30. pH was adjusted to 6.4 using KOH and buffered with 0.25 mM 2-(N-morpholino) ethane-sulfonic acid (MES). After transfer to the growth boxes, the nutrient solution was adjusted once to a 0.5 mM NH_4_^+^ concentration by the addition of (NH_4_)_2_SO_4_ since low concentrations of ammonium support growth and nodule formation in young *M. truncatula* plants^45^. During their growth in the glass tubes, the plants depended solely on nitrogen fixation for their N nutrition. Phosphorus (P) was added daily as KH_2_PO_4_ to the nutrient solution to adjust a concentration of 5 μM P for plants in the control and the nitrate experiment. The nutrient solution in the low-P treatment was adjusted daily to 1 μM P.

The nutrient solution was changed every week. During this procedure, the pump in the container was switched off and the backflow from the glass tubes to the container was blocked. Using this method, the ongoing measurements in the root/nodule compartment were not affected. The procedure of replacing the nutrient solution in the container took about ten minutes, after which the nutrient solution turnover system was set back to normal.

### Dry matter, P and N concentration in plant material

Plants were dried to constant weight at 65 °C before the dry matter was determined. Subsamples of plant dry matter were digested in concentrated HNO_3_ at 180 °C and the P concentration in the digest was measured colorimetrically by the molybdenum-vanadate method^46^. N concentration was determined by means of an elementary analyser (Vario EL, Elementar Analysen GmbH, Hanau, Germany)^47^.

### Root/nodule gas exchange measurement

The system for measuring nodule H_2_ evolution, including the determination of apparent nitrogenase activity (ANA), total nitrogenase activity (TNA) and the calculation of the electron allocation coefficient (EAC), is described by Fischinger and Schulze^47^. ANA was measured continuously over a two-day period. The H_2_ concentration data in the continuous gas stream were taken every 5 min. TNA was measured on parallel plants at various points in time during the experiment.

### Quantitative reverse transcription-polymerase chain reaction (qRT-PCR)

For the quantitative reverse transcription-polymerase chain reaction (qRT-PCR), gene-specific primers were designed using Primer-Blast (http://www.ncbi.nlm.nih.gov/tools/primer-blast). To validate the RNAseq results, RNA from the RNA pools previously used for Illumina sequencing was used. The samples and reference genes were run in triplicate using the *Fast SYBR Green Master Mix* protocol (Applied Biosystems, Darmstadt, Germany) on a StepOne™ Real-Time PCR System (Applied Biosystems, Darmstadt, Germany) following the manufacturer’s recommendations.

qRT-PCR was performed in a 20-μL reaction mix containing 10 μL *Fast SYBR Green Master Mix*, 4 μL ddH_2_O, 2 μL forward primer (2 pmol/μL), 2 μL reverse primer (2 pmol/μL) and 2 μL template cDNA (100ng). The PCR conditions according to the *Fast SYBR Green Master Mix* protocol were 20 seconds of pre-denaturation at 95 °C, 45 cycles of 3 seconds at 95 °C and 30 sec at 60 °C, followed by steps for the dissociation curve generation (15 sec at 95 °C, 60 sec at 60 °C and a stepwise increase of 0.3 °C up to 95 °C). StepOnePlus software (Applied Biosystems, CA, USA) was used for data collection. Relative transcript levels were obtained using the comparative C_T_ method (ΔΔC_T_).

### RNA extraction, cDNA library preparation and RNAseq

Total RNA was purified from 27 samples [(untreated controls, 4 h nitrate, P-deficiency) × (three biological replicates) × (nodules from three plants to be pooled for one biological treatment)] using the TRIzol protocol (Invitrogen, Frankfurt, Germany). RNA was digested with RNAse-free DNAse (Qiagen, Hilden, Germany) and checked for integrity by capillary gel electrophoresis (Bioanalyzer, Agilent Technologies, Inc., Böblingen, Germany).

Library preparation for RNAseq was performed using the TruSeq RNA Sample Preparation Kit (Illumina, Cat. N°RS-122-2002) starting from 500 ng of total RNA. Accurate quantitation of cDNA libraries was performed using the QuantiFluor™ dsDNA System (Promega, Mannheim, Germany). The size range of the final cDNA libraries was determined by applying the DNA 1000 chip on the Bioanalyzer 2100 from Agilent (Böblingen, Germany) (280 bp). The cDNA libraries were amplified and sequenced using the cBot and HiSeq2000 from Illumina (SR; 1 × 50 bp; 6 GB ca. 30–35 million reads per sample).

The sequence images were transformed with the Illumina software BaseCaller to bcl-files, which were demultiplexed to fastq files with CASAVA v1.8.2. Quality check of raw data was performed with the FastQC Software (v.0.10.0, Babraham Bioinformatics, Cambridge, UK), in order to assess the overall and per-base quality of reads. Illumina adapters were removed with the Trimmomatic Software^48^. The sequences were aligned by Bowtie2 (v2.0.2, Johns Hopkins University, Baltimore, Maryland, USA) to the J. Craig Venter Institute (JCVI) cds annotation Mt4.0v1.

### Gene expression and statistical analyses

The expression level of TUs in each library was calculated by quantifying the number of Illumina reads that mapped to the Mt4.0v1 cds annotation using the Bowtie program, counting only ‘unique hits’. The term ‘gene expression’ or ‘expression level of the TU’ is used, although what is measured by RNAseq is a net result of expression and decay intensity of mRNA. The raw counts were normalised using the RPKM method^49^. RPKM values were used to compare the abundance of TUs within one treatment. Transcript units showing differential abundance were identified using the DESeq method for pair-wise differential expression analysis since this has proven to be the most reliable method for RNAseq based differential expression analysis^50^. Differentially abundant transcript units identified by DESeq were required to have an FDR value < 0.01. Additionally, either the control or treatment tissues had to have a DESeq value above 20 before a gene was considered as having been expressed. Figures 1, 2 and 5 were generated using GraphPad Prism 6. Statistical differences among treatments were calculated using a *t*-test. Cluster analysis for the dendrogram (Fig. 3) was calculated with the R package “hclust” through a complete-linkage clustering. The Venn diagram was generated for the set of TUs differentially expressed in both treatments at http://bioinformatics.psb.ugent.be/webtools/Venn/.

## Additional Information

**How to cite this article**: Liese, R. *et al*. Nitrate application or P deficiency induce a decline in *Medicago truncatula* N_2_-fixation by similar changes in the nodule transcriptome. *Sci. Rep.* **7**, 46264; doi: 10.1038/srep46264 (2017).

**Publisher's note:** Springer Nature remains neutral with regard to jurisdictional claims in published maps and institutional affiliations.

## Supplementary Material

Supplementary Dataset 1

Supplementary Dataset 2

Supplementary Dataset 3

Supplementary Dataset 4

## Figures and Tables

**Table 1:** Dry matter (DM) and per day specific activity of nodules.

	Control		Low P	
	active	inactive	active	inactive
Nodule DM [mg]	180.7	39.4	33	28.6
Nodule specific activity [μg N_fixed_ mg DM active nodule^−1^ d^−1^]	49.2	—	31.8	—
Nodule specific activity [μg N_fixed_ mg DM nodule^−1^ d^−1^]	40.4		17.1	

Data were taken from six biological replicates. N reduction was calculated from a continuous measurement of nodule H_2_ evolution (ANA) and the point measurement of H_2_ evolution in an Ar/O_2_ mixture (79/21, v/v) (TNA). The N_2_ reduction per day was calculated under the assumption of a constant electron allocation between H^+^ and N_2_.

## References

[b1] Schulze J., Adgo E. & Merbach W. Carbon costs associated with N_2_ fixation in *Vicia faba* L and *Pisum sativum* L over a 14-day period. Plant Biol. 1, 625–631 (1999).

[b2] Ryle G. J. A., Powell C. E. & Gordon A. J. The respiratory costs of nitrogen fixation in soyabean, cowpea, and white clover II. Comparisons of the cost of nitrogen fixation and the utilization of combined nitrogen. J. Exp. Bot. 30, 145–153 (1979).

[b3] Ferguson B. J. . Molecular analysis of legume nodule development and autoregulation. J. Integr. Plant Biol. 52, 61–76 (2010). 20074141 10.1111/j.1744-7909.2010.00899.x

[b4] Reid D. E., Ferguson B. J., Hayashi S., Lin Y.-H. & Gresshoff P. M. Molecular mechanisms controlling legume autoregulation of nodulation. Ann. Bot. 108, 789–795 (2011). 21856632 10.1093/aob/mcr205 PMC3177682

[b5] Hayashi S., Gresshoff P. M. & Ferguson B. J. Mechanistic action of gibberellins in legume nodulation. J. Integr. Plant Biol. 56, 971–978 (2014). 24673766 10.1111/jipb.12201

[b6] Salon C. . Analysis and modeling of the integrative response of *Medicago truncatula* to nitrogen constraints. C. R. Biol. 332, 1022–1033 (2009). 19909924 10.1016/j.crvi.2009.09.009

[b7] Voisin A.-S., Munier-Jolain N. G. & Salon C. The nodulation process is tightly adjusted to plant growth. An analysis using environmentally and genetically induced variation of nodule number and biomass in pea. Plant Soil 337, 399–412 (2010).

[b8] Becana M., Minchin F. R. & Sprent J. I. Short-term inhibition of legume N_2_ fixation by nitrate. I. Nitrate effects on nitrate-reductase activities of bacteroids and nodule cytosol. Planta 180, 40–45 (1989). 24201842 10.1007/BF02411408

[b9] Gil-Quintana E. . Local inhibition of nitrogen fixation and nodule metabolism in drought-stressed soybean. J. Exp. Bot. 64, 2171–2182 (2013). 23580751 10.1093/jxb/ert074 PMC3654410

[b10] Cabeza R. A. . RNA-seq transcriptome profiling reveals that *Medicago truncatula* nodules acclimate N_2_ fixation before emerging P deficiency reaches the nodules. J. Exp. Bot. 65, 6035–6048 (2014). 25151618 10.1093/jxb/eru341 PMC4203135

[b11] Bacanamwo M. & Harper J. E. The feedback mechanism of nitrate inhibition of nitrogenase activity in soybean may involve asparagine and/or products of its metabolism. Physiol. Plant. 100, 371–377 (1997).

[b12] Hartwig U. A. The regulation of symbiotic N_2_ fixation: a conceptual model of N feedback from the ecosystem to the gene expression level. Perspect. Plant Ecol. Evol. Syst. 1, 92–120 (1998).

[b13] Cabeza R. A. . Long-term non-invasive and continuous measurements of legume nodule activity. Plant J. 81, 637–648 (2015). 25640854 10.1111/tpj.12751

[b14] Streeter J. & Wong P. P. Inhibition of legume nodule formation and N_2_ fixation by nitrate. Crit. Rev. Plant Sci. 7, 1–23 (1988).

[b15] Naudin C. . Inhibition and recovery of symbiotic N_2_ fixation by peas (*Pisum sativum* L.) in response to short-term nitrate exposure. Plant Soil 346, 275–287 (2011).

[b16] Cabeza R. A. . An RNA sequencing transcriptome analysis reveals novel insights into molecular aspects of the nitrate impact on the nodule activity of *Medicago truncatula*. Plant Physiol. 164, 400–411 (2014). 24285852 10.1104/pp.113.228312 PMC3875817

[b17] Bhadoria P. S., Dessougi H. E., Liebersbach H. & Claassen N. Phosphorus uptake kinetics, size of root system and growth of maize and groundnut in solution culture. Plant Soil 262, 327–336 (2004).

[b18] Vance C. P. Symbiotic nitrogen fixation and phosphorus acquisition. Plant nutrition in a world of declining renewable resources. Plant Physiol. 127, 390–397 (2001). PMC1540145 11598215

[b19] Schulze J., Temple G., Temple S. J., Beschow H. & Vance C. P. Nitrogen fixation by white lupin under phosphorus deficiency. Ann. Bot. 98, 731–740 (2006). 16855013 10.1093/aob/mcl154 PMC2806177

[b20] Almeida J. F., Hartwig U. A., Frehner M., Nösberger J. & Lüscher A. Evidence that P deficiency induces N feedback regulation of symbiotic N_2_ fixation in white clover (*Trifolium repens* L.). J. Exp. Bot. 51, 1289–1297 (2000). 10937705

[b21] Høgh-Jensen H., Schjoerring J. K. & Soussana J.-F. The influence of phosphorus deficiency on growth and nitrogen fixation of white clover plants. Ann. Bot. 90, 745–753 (2002). 12451030 10.1093/aob/mcf260 PMC4240371

[b22] Avenhaus U. . Short-term molecular acclimation processes of legume nodules to increased external oxygen concentration. Front. Plant Sci. 1133, doi: 10.3389/fpls.2015.01133 (2016). PMC4702478 26779207

[b23] Tang C., Hinsinger P., Drevon J. J. & Jaillard B. Phosphorus deficiency impairs early nodule functioning and enhances proton release in roots of *Medicago truncatula* L. Ann. Bot. 88, 131–138 (2001).

[b24] Sulieman S., Fischinger S. A., Gresshoff P. M. & Schulze J. Asparagine as a major factor in the N-feedback regulation of N_2_ fixation in *Medicago truncatula*. Physiol. Plant. 140, 21–31 (2010). 20444196 10.1111/j.1399-3054.2010.01380.x

[b25] Schulze J. & Drevon J.-J. P-deficiency increases the O_2_ uptake per N_2_ reduced in alfalfa. J. Exp. Bot. 56, 1779–1784 (2005). 15851413 10.1093/jxb/eri166

[b26] Brear E. M., Day D. A. & Smith P. M. C. Iron: an essential micronutrient for the legume–rhizobium symbiosis. Front. Plant Sci. 4, 359 (2013). 24062758 10.3389/fpls.2013.00359 PMC3772312

[b27] Curie C. . Metal movement within the plant: contribution of nicotianamine and yellow stripe 1-like transporters. Ann. Bot. 103, 1–11 (2009). 18977764 10.1093/aob/mcn207 PMC2707284

[b28] Kobayashi T. & Nishizawa N. K. Iron uptake, translocation, and regulation in higher plants. Annu. Rev. Plant Biol. 63, 131–152 (2012). 22404471 10.1146/annurev-arplant-042811-105522

[b29] Van de Velde W. . Plant peptides govern terminal differentiation of bacteria in symbiosis. Science 327, 1122–1126 (2010). 20185722 10.1126/science.1184057

[b30] Becker A. . Global changes in gene expression in *Sinorhizobium meliloti* 1021 under microoxic and symbiotic conditions. Mol. Plant Microbe Interact. 17, 292–303 (2004). 15000396 10.1094/MPMI.2004.17.3.292

[b31] Oono R., Anderson C. G. & Denison R. F. Failure to fix nitrogen by non-reproductive symbiotic rhizobia triggers host sanctions that reduce fitness of their reproductive clonemates. Proc. R. Soc. Lond. B Biol. Sci. 278, 2698–2703 (2011). 10.1098/rspb.2010.2193 PMC3136820 21270038

[b32] Oono R., Denison R. F. & Kiers E. T. Controlling the reproductive fate of rhizobia: how universal are legume sanctions? New Phytol. 183, 967–979 (2009). 19594691 10.1111/j.1469-8137.2009.02941.x

[b33] Horváth B. . Loss of the nodule-specific cysteine rich peptide, NCR169, abolishes symbiotic nitrogen fixation in the *Medicago truncatula* dnf7 mutant. Proc. Natl. Acad. Sci. USA 112, 15232–15237 (2015). 26401023 10.1073/pnas.1500777112 PMC4679056

[b34] Kim M. . An antimicrobial peptide essential for bacterial survival in the nitrogen-fixing symbiosis. Proc. Natl. Acad. Sci. USA 112, 15238–15243 (2015). 26598690 10.1073/pnas.1500123112 PMC4679048

[b35] Nallu S. . Regulatory patterns of a large family of defensin-like genes expressed in nodules of *Medicago truncatula*. PLoS ONE 8, e60355 (2013). 23573247 10.1371/journal.pone.0060355 PMC3613412

[b36] Wang D. . A nodule-specific protein secretory pathway required for nitrogen-fixing symbiosis. Science 327, 1126–1129 (2010). 20185723 10.1126/science.1184096 PMC4824053

[b37] Appleby C. A. Leghemoglobin and rhizobium respiration. Ann. Rev. Plant Physiol. 35, 443–478 (1984).

[b38] Denison R. F. & Harter B. L. Nitrate effects on nodule oxygen permeability and leghemoglobin (nodule oximetry and computer modeling). Plant Physiol. 107, 1355–1364 (1995). 12228439 10.1104/pp.107.4.1355 PMC157270

[b39] Ribet J. & Drevon J.-J. Increase in permeability to oxygen and in oxygen uptake of soybean nodules under limiting phosphorus nutrition. Physiol. Plant. 94, 298–304 (1995).

[b40] Ott T. . Symbiotic leghemoglobins are crucial for nitrogen fixation in legume root nodules but not for general plant growth and development. Curr. Biol. 15, 531–535 (2005). 15797021 10.1016/j.cub.2005.01.042

[b41] Downie J. A. Legume haemoglobins: symbiotic nitrogen fixation needs bloody nodules. Curr. Biol. 15, R196–R198 (2005). 15797009 10.1016/j.cub.2005.03.007

[b42] Blumenthal J. M., Russelle M. P. & Vance C. P. Nitrogenase activity is affected by reduced partial pressures of N_2_ and NO_3_^−^. Plant Physiol. 114, 1405–1412 (1997). 12223779 10.1104/pp.114.4.1405 PMC158433

[b43] Fischinger S. A. & Schulze J. The argon-induced decline in nitrogenase activity commences before the beginning of a decline in nodule oxygen uptake. J. Plant Physiol. 167, 1112–1115 (2010). 20488580 10.1016/j.jplph.2010.03.014

[b44] Fischinger S. A., Hristozkova M., Mainassara Z.-A. & Schulze J. Elevated CO_2_ concentration around alfalfa nodules increases N_2_ fixation. J. Exp. Bot. 61, 121–130 (2010). 19815686 10.1093/jxb/erp287 PMC2791116

[b45] Fei H. & Vessey J. K. Stimulation of nodulation in *Medicago truncatula* by low concentrations of ammonium: quantitative reverse transcription PCR analysis of selected genes. Physiol. Plant. 135, 317–330 (2009). 19140888 10.1111/j.1399-3054.2008.01192.x

[b46] Scheffer F. & Pajenkamp H. Phosphatbestimmung in Pflanzenaschen nach der Molybdän-Vanadin-Methode. Journal of Plant Nutrition and Soil Science 56, 2–8 (1952).

[b47] Fischinger S. A. & Schulze J. The importance of nodule CO_2_ fixation for the efficiency of symbiotic nitrogen fixation in pea at vegetative growth and during pod formation. J. Exp. Bot. 61, 2281–2291 (2010). 20363863 10.1093/jxb/erq055 PMC2877887

[b48] Bolger A. M., Lohse M. & Usadel B. Trimmomatic: a flexible trimmer for Illumina sequence data. Bioinformatics 30, 2114–2120 (2014). 24695404 10.1093/bioinformatics/btu170 PMC4103590

[b49] Mortazavi A., Williams B. A., McCue K., Schaeffer L. & Wold B. Mapping and quantifying mammalian transcriptomes by RNA-Seq. Nat. Methods 5, 621–628 (2008). 18516045 10.1038/nmeth.1226 PMC13303166

[b50] Dillies M.-A. . A comprehensive evaluation of normalization methods for Illumina high-throughput RNA sequencing data analysis. Brief. Bioinform. 14, 671–683 (2013). 22988256 10.1093/bib/bbs046

